# Obstructive sleep apnea risk and its association with diabetic foot ulcer in patients with type 2 diabetes

**DOI:** 10.3389/fendo.2026.1813327

**Published:** 2026-05-29

**Authors:** Raquel Ruano, María Teresa Charles, Josep León-Mengíbar, Ferran Barbé, Angel Michael Ortiz-Zúñiga, Marta Hernández, Cristina Hernández, Rafael Simó, Albert Lecube

**Affiliations:** 1 Department of Endocrinology and Nutrition, University Hospital Arnau de Vilanova de Lleida, Obesity, Diabetes and Metabolism (ODIM) Research Group, Institute for Research in Biomedicine of Lleida (IRBLleida), University of Lleida, Lleida, Spain; 2 Translational Research in Respiratory Medicine, University Hospital Arnau de Vilanova and Santa Maria, Institute for Research in Biomedicine of Lleida (IRBLleida), CIBER of Respiratory Diseases (CIBERES), Institute of Health Carlos III, Lleida, Spain; 3 Department Endocrinology and Nutrition, University Hospital Vall d’Hebron. Diabetes and Metabolism Research Group, Vall d’Hebron Research Institute (VHIR), CIBERDEM (Institute of Health Carlos III), Barcelona, Spain

**Keywords:** amputation risk, diabetic foot ulcer, obstructive sleep apnea, STOP-Bang questionnaire, type 2 diabetes mellitus, WIfI classification

## Abstract

**Background:**

Obstructive sleep apnea (OSA) is common in type 2 diabetes (T2D) and may contribute to its chronic complications. Whether STOP-Bang-defined OSA risk is associated with diabetic foot ulcer (DFU) severity and outcome remains unclear. We compared STOP-Bang score between T2D patients with and without DFU and explored the association between OSA risk and DFU severity and healing.

**Methods:**

We conducted an observational study with a cross-sectional matched case-control comparison including 73 T2D patients with DFU and 73 T2D controls without DFU, matched by age, HbA1c, BMI and smoking. At the study visit, STOP-Bang-defined OSA risk was assessed as a continuous score and categorized as low versus moderate/high risk. In the DFU group, ulcer severity was graded at the same visit with the WIfI classification, and time to healing and amputation was recorded during clinical follow-up. Multivariable logistic regression identified predictors of moderate-to-high amputation risk.

**Results:**

In unadjusted analyses, DFU patients had higher STOP-Bang score than controls (4.3 ± 2.0 vs. 3.2 ± 2.2, p=0.004) and more often intermediate/high OSA risk (83.6% vs. 60.3%, p=0.002), although these differences were attenuated after accounting for sex imbalance. Among DFU patients, higher ulcer severity was associated with moderate/high STOP-Bang-defined OSA risk (97.1 vs. 71.8%; p=0.004). Median healing time was longer in those with moderate/high versus low STOP-Bang-defined OSA risk (120.0 vs. 65.0 days, p=0.032). In multivariable analysis, WIfI ulcer severity was associated with male sex, higher STOP-Bang category and poorer metabolic control.

**Conclusion:**

STOP-Bang-defined OSA risk was associated with greater DFU severity and delayed wound healing. Incorporating OSA risk stratification into routine assessment of patients with DFU may help identify individuals at higher risk of poor outcomes.

## Introduction

Obstructive sleep apnea (OSA) is characterized by recurrent upper-airway collapse during sleep, leading to periodic reductions in ventilation and sustained–intermittent hypoxemia, which may impair tissue oxygenation in chronic wounds and delay healing (1). Various risk factors contribute to OSA development, some nonmodifiable (such as male sex, increasing age, and craniofacial anatomy leading to narrow airways) and others modifiable, including obesity, medications that cause muscle relaxation and airway narrowing (e.g., opiates, benzodiazepines, alcohol), endocrine disorders (e.g., hypothyroidism, polycystic ovary syndrome), and smoking (2, 3). Increasing evidence also suggests that type 2 diabetes (T2D) may contribute to OSA pathogenesis, heightening the risk of severe nocturnal hypoxemia, nocturnal awakenings, and excessive daytime sleepiness (1, 4–6). Despite the high burden of disease, OSA remains widely underdiagnosed and undertreated, with an estimated global prevalence of approximately 50% (7, 8).

Sleep-related hypoxia triggers oxidative stress, sympathetic activation, and endothelial dysfunction, mechanisms that overlap with those driving chronic microvascular and macrovascular complications in T2D (9–11). Consequently, it is biologically plausible that sleep-disordered breathing and underdiagnosed OSA could worsen peripheral tissue hypoxia in patients with T2D, thereby contributing to the development, progression, and impaired healing of complications such as diabetic foot ulcers (DFU) (12). Globally, DFU affects approximately 18.6 million individuals each year, precedes 80% of lower-extremity amputations, and is associated with increased mortality, representing one of the most serious and potentially life-threatening complications in people with T2D (13, 14). Of note, the prevalence of severe OSA (apnea–hypopnea index ≥15 events/hour) in patients with DFU has been reported to range between 55% and 57%, clearly exceeding that observed in the general middle-aged population (1, 6). However, whether nocturnal hypoxemia in patients with DFU further compromises distal tissue oxygenation, leading to more severe lesions and delayed healing, remains unclear, underscoring the need for dedicated studies to elucidate this potential interaction. Insulin resistance and chronic hyperglycemia may contribute to OSA through mechanisms such as pharyngeal neuropathy, upper-airway fat deposition, and ventilatory instability, establishing a bidirectional relationship between metabolic dysfunction and sleep-disordered breathing (5, 15). Moreover, patients with T2D demonstrate abnormal and blunted ventilatory responses to isocapnic hypoxia, suggesting an impaired homeostatic response to hypoxic stress. Metabolic pathways related to leptin resistance in diabetes may further impair central respiratory control in patients with T2D (16–19).

Despite these potential links, evidence on the impact of OSA or OSA risk in patients with DFU remains scarce. Moreover, it is still uncertain whether a higher risk of OSA, as assessed by simple screening tools such as the STOP-Bang questionnaire, is more frequent in patients with DFU than in other individuals with T2D, and whether it is associated with ulcer severity and a delay in wound healing (20–24). Therefore, the aims of the present study were: (i) to compare OSA risk, estimated by the STOP-Bang score, between patients with T2D with and without DFU; and (ii) among patients with DFU, to evaluate the association between STOP-Bang–defined OSA risk and ulcer severity and time to ulcer healing.

## Methodology

### Ethics statement

Written informed consent was obtained from all participants, and the Institutional Ethics Committee of Arnau de Vilanova University Hospital (Lleida, Spain) approved the study protocol and the consent procedure, in accordance with the Declaration of Helsinki (CEIC-2617). No identifiable individual participant data or images are reported in this manuscript.

### Main association

The main association of interest was between STOP-Bang–defined OSA risk and the presence, clinical characteristics, and short-term prognosis (healing time and amputation) of diabetic foot ulcers.

### Design of the study

This was an observational study with two complementary analyses. First, we performed a cross-sectional matched case-control comparison of STOP-Bang-defined OSA risk between 73 patients with T2D and active DFU and 73 matched patients with T2D without current or previous DFU. Cases and controls were matched for age, BMI, and HbA1c, but not for sex. In patients with DFU, the STOP-Bang questionnaire was administered at the first visit to the Diabetic Foot Clinic, after diagnosis of the active ulcer. Controls were recruited among patients with T2D attending the Endocrinology outpatient clinic for routine diabetes follow-up, where the STOP-Bang questionnaire was administered. Both cases and controls were recruited at Arnau de Vilanova University Hospital (Lleida, Spain) between September 2023 and September 2024.

In the second part of the study, among patients with active DFU, we assessed the cross-sectional association between baseline STOP-Bang category, defined as moderate/high versus low OSA risk, and ulcer severity, as defined by the WIfI classification. Time to healing was subsequently recorded during routine clinical follow-up and analyzed exploratorily according to baseline STOP-Bang category. The study followed the STROBE criteria for reporting observational studies (25).

To reduce selection bias, we applied identical inclusion and exclusion criteria to DFU cases and T2D controls and recruited both groups within the same hospital during the same time frame. Information bias was minimized by administering the STOP-Bang questionnaire in a standardized manner at the first visit and by using the WIfI classification to grade ulcer severity. Residual confounding cannot be excluded, particularly for variables not included in the matching or multivariable models.

### Sample size calculation

The sample size was primarily determined by feasibility within a single-center Diabetic Foot Clinic (DF Clinic). Based on previous studies, the prevalence of high STOP-Bang-defined OSA risk (STOP-Bang ≥5) was expected to be around 49.3% to 58.7% in patients with T2D and approximately 64% in those with T2D and DFU, yielding an absolute difference of 5–15 percentage points between groups (21, 26, 27). Under these assumptions, detecting such differences with 80% power and a two-sided α=0.05 would require several hundred participants per group, which was not feasible in this setting. Therefore, the case-control sample size was based on feasibility, and STOP-Bang was analyzed primarily as a continuous score and as an exploratory categorical variable. A sample of approximately 60–70 participants per group was considered sufficient to detect a moderate standardized difference in STOP-Bang score (Cohen’s d≈0.5). Ultimately, 73 patients with DFU and 73 matched T2D controls were enrolled.

For the exploratory analysis within the DFU group, the number of predictors in the multivariable logistic regression model was restricted because of the modest sample size (28). Six clinically relevant covariates were selected *a priori* and entered simultaneously into the model. The model should therefore be considered exploratory.

### Description of patients evaluated for risk of having OSA

A total of 143 Caucasian patients attending the outpatient DF Clinic at the Arnau de Vilanova University Hospital were enrolled at their first visit for an active foot ulcer between September 2023 and September 2024. Inclusion criteria were: age >18 years, a diagnosis of T2D for more than 5 years, presence of polyneuropathy, and availability of a glycated hemoglobin (HbA1c) measurement within the previous month. Of the 118 patients with T2D and an active DFU who met these criteria, 36 were excluded for the following reasons: missing data (n = 7), chronic pulmonary disease (n = 3), previous diagnosis of OSA (n = 7) or treatment with continuous positive airway pressure (CPAP) (n = 5), stroke or heart failure (n = 2), alcohol abuse or use of sedatives (n = 2), active malignancy (n = 4), referral for Charcot arthropathy without DFU (n = 3), institutionalization (n = 2), end-stage renal disease (n = 2), and clinical manifestations of diabetic autonomic neuropathy (n = 1). No pregnant women or shift workers were included in the study. Among patients with T2D without DFU, we aimed to select one control for each case. The control group therefore consisted of 73 subjects attending the outpatient clinic of the Endocrinology and Nutrition Department, individually matched to cases by age ( ± 3 years), BMI ( ± 2.0 kg/m²), metabolic control (HbA1c ±1.0%), and smoking status. Diabetic polyneuropathy was diagnosed based on symptoms and signs (neuropathic symptoms, decreased vibration, pressure, or pinprick sensation) using standard bedside tests. Peripheral artery disease was defined by abnormal ankle–brachial index (<0.9 or >1.3), toe pressure <70 mmHg, or prior lower-limb revascularization.

### Specific questionnaire to assess risk of OSA

The STOP-Bang questionnaire was used as a screening instrument to estimate the likelihood of OSA, not to establish a clinical diagnosis. It is a simple, easy-to-use and validated screening tool comprising eight dichotomous (yes/no) items: snoring (S), tiredness (T), observed apneas (O), high blood pressure (P), body mass index >35 kg/m² (B), age > 50 years (A), neck circumference > 40cm (N), and male sex (G) (29, 30). Each affirmative answer scores one point, yielding a total score ranging from 0 to 8. For the purposes of this study, OSA risk was classified as low (0–2 positive responses), moderate (3–4 positive responses), or high (≥ 5 positive responses, or ≥2 positive responses to the first four questions combined with male sex, body mass index >35 kg/m², and/or neck circumference >40cm). No participant underwent polysomnography or any sleep study confirmation as part of the study protocol.

### Diabetic foot ulcer assessment

DFU was defined as a full-thickness lesion of the skin distal to the malleoli persisting for ≥2 weeks. DFU characteristics were recorded, including ulcer size, depth, presence of inflammation, infection severity, ischemia, time to healing, recurrence, and need for amputation. Ulcers were classified according to the Society for Vascular Surgery’s WIfI (*Wound, Ischemia, and foot Infection*) classification system, which grades each component from 0 (least severe) to 3 (most severe): wound (extent and depth of ulceration or presence of gangrene), ischemia (based on ankle–brachial index, toe pressure, or transcutaneous oxygen pressure), and foot infection (severity according to clinical findings and Infectious Diseases Society of America (IDSA) criteria (31, 32). The three grades are combined to stratify 1-year amputation risk and to estimate the potential benefit of revascularization. Assessment included clinical evaluation, peripheral artery disease screening, and imaging studies when indicated (from plain radiographs to magnetic resonance imaging).

### Statistical analysis

The normality of the variables was assessed using the Kolmogorov–Smirnov test. Data were expressed as mean ± SD, percentage, or median (total range). Comparisons between groups were performed using the Student’s t test or the Mann–Whitney U test for continuous variables, and the χ² test for categorical variables. For statistical purposes, STOP-Bang scores were grouped into two categories: low risk (0–2 points) and moderate-to-high risk (≥3 points) of having OSA, according to established cut-offs (29, 30). Similarly, WIfI scores were grouped into lower risk of amputation (stage 1) and moderate-to-high risk of amputation (stages 2 and 3), following the Society for Vascular Surgery classification (31). These categorizations were applied to simplify the analyses and because of the limited number of patients in some of the original categories. Analyses were conducted using complete-case data; no imputation of missing values was performed. Data were completed for all variables included in the main analyses, except for diabetes duration, which was available for 114 participants overall, including 49 patients with DFU and 65 controls. Because of this incomplete availability, diabetes duration was analyzed descriptively but was not included in the multivariable model.

Given the marked imbalance in sex distribution between DFU cases and controls, and because male sex is one of the STOP-Bang items, two additional sensitivity analyses were performed for the case-control comparison. First, we assessed the association between DFU status and STOP-Bang category using logistic regression adjusted for sex. Second, we calculated a modified STOP-Bang score excluding the sex item, ranging from 0 to 7, and compared this score between DFU cases and controls.

Additionally, among patients with DFU, a multiple binary logistic regression model was fitted to identify variables independently associated with the moderate-to-high risk to amputation according the WIfI score. To limit overfitting given the available sample size, we pre-specified a maximum of six candidates on the basis of clinical relevance, prior evidence, and their availability in the dataset, and were entered simultaneously into the model. The final model included STOP-bang category, HbA1c, age, sex, peripheral artery disease, and previous DFU. These variables were selected because they represent established or clinically plausible determinants of ulcer severity and/or OSA risk (33, 34). Diabetic polyneuropathy was not included because it was almost universal in the DFU cohort, limiting its discriminatory value. BMI was not included because it is a component of the STOP-Bang score and has already been used as a matching variable in the case-control comparison. Diabetes duration was not included because of incomplete data, and no significant differences were observed across STOP-Bang categories in the available data. Model fit was evaluated using deviance statistics and pseudo-R² measures.

Time to ulcer healing was analyzed as an exploratory outcome by comparing median healing time between STOP-Bang categories using non-parametric tests. Kaplan-Meier curves and Cox regression analyses were not performed. All p values were two-sided, with statistical significance set at p<0.05. Analyses were performed using the jamovi statistical package (The jamovi project, version 2.6.5).

Finally, the discriminative ability of the final multivariate logistic regression model for predicting the risk of amputation was assessed using the area under the receiver operating characteristic curve (AUC-ROC) with its 95% confidence interval (CI). The AUC-ROC was interpreted as follows: 0.5–0.6 = fail, 0.6–0.7 = poor, 0.7–0.8 = acceptable, 0.8–0.9 = excellent, and >0.9 = outstanding discrimination (35).

## Results

A total of 146 participants with T2D were included: 73 patients with an active foot ulcer and 73 attending general outpatient clinics (control group) (**Table 1**). Compared with controls, the group attending the Diabetic Foot Unit had a markedly higher prevalence of previous ulcers (65.8% vs. 1.4%, p<0.001) and prior amputation (46.6% vs. 1.4%, p<0.001) (**Table 1**). They also had a higher proportion of men (86.3% vs. 42.5%, p<0.001), along with a substantially greater burden of microvascular and macrovascular complications.

**Table 1:** Main clinical and metabolic characteristics of study participants according to the presence of diabetic foot ulcer (DFU).

Variables	DFU group	Control group	p
n	73	73	-
Age (years)	65.9 ± 8.8	63.6 ± 12.4	0.203
BMI (kg/m²)	29.9 ± 6.8	29.5 ± 7.1	0.731
HbA1c (%)	7.7 ± 1.8	7.7 ± 1.4	0.913
Duration of T2D (years) *	15.9 ± 11.0	15.1 ± 10.3	0.681
Former smoker, n (%)	11 (15.7)	11 (15.7)	0.915
Men, n (%)	63 (86.3)	31 (42.5)	<0.001
Previous DFU, n (%)	48 (65.8)	1 (1.4)	<0.001
Previous amputation, n (%)	34 (46.6)	1 (1.4)	<0.001
Retinopathy, n (%)	40 (54.8)	21 (28.8)	0.001
Polyneuropathy, n (%)	73 (100.0)	15 (20.5)	<0.001
Peripheral artery disease, n (%)	35 (47.9)	2 (2.7)	<0.001
STOP-Bang score	4.3 ± 2.0	3.2 ± 2.2	0.004
Moderate-to-high STOP-Bang score, n (%)	61 (83.6)	44 (60.3)	0.002

Values are presented as mean ± standard deviation or n (%). *Information on T2D duration was available for 114 patients (49 with DFU and 65 controls). BMI, body mass index; HbA1c, glycated hemoglobin; T2D, type 2 diabetes; DFU, diabetic foot ulcer.

**Figure 1:**
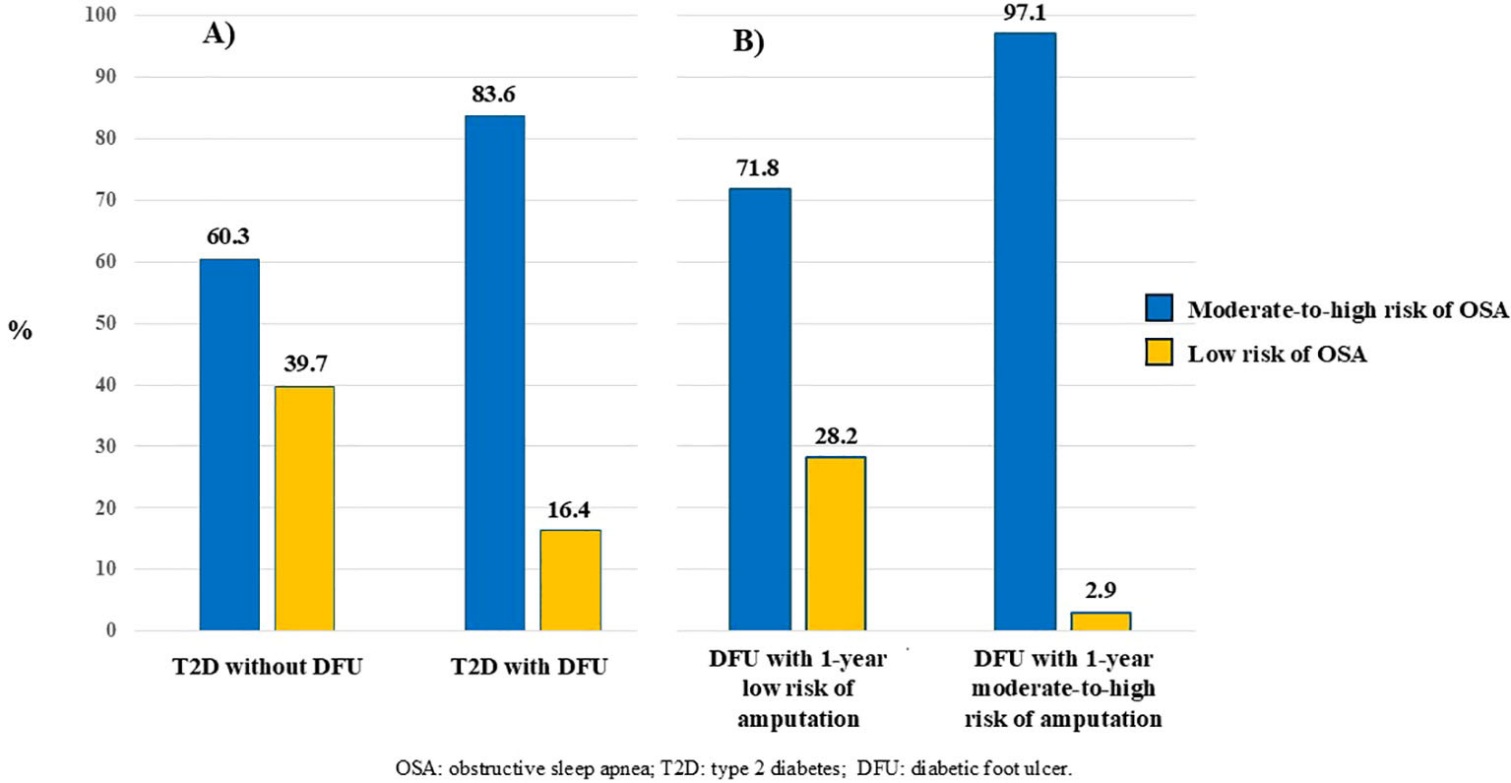
Risk of obstructive sleep apnea according to the STOP-Bang score in **(A)** patients with type 2 diabetes stratified by the presence of an active diabetic foot ulcer, and **(B)** patients with diabetic foot ulcer stratified by 1-year amputation risk (WIfI classification).

Regarding the STOP-Bang score, both the total score and the prevalence of participants with moderate-to-high STOP-Bang-defined OSA risk was significantly higher among DFU group in comparison with the control group (83.6% vs. 60.3%, p=0.002) (**Figure 1**). However, in sensitivity analyses accounting for the marked sex imbalance between groups, this association was attenuated. In logistic regression with DFU status as the dependent variable, moderate-to-high STOP-Bang–defined OSA risk was no longer significantly associated with DFU after adjustment for sex (OR 1.44, 95% CI 0.57-3.63; p=0.437), whereas female sex was associated with lower odds of DFU compared with male sex (OR 0.13, 95% CI 0.05-0.32; p<0.001). In addition, when a modified STOP-Bang score excluding the sex item was compared between groups, patients with DFU showed numerically higher scores than controls, although the difference was no longer statistically significant (3.41 ± 1.94 vs. 2.82 ± 2.08; p=0.078).

Considering the entire population, moderate-to-high STOP-Bang scores were strongly associated with male sex, older age, and higher BMI, showing also greater prevalence of peripheral artery disease, polyneuropathy, previous DFU, and previous amputation (**Table 2**). However, no differences in metabolic control or known T2D duration were observed between STOP-Bang categories.

**Table 2:** Main clinical and metabolic characteristics of the entire population stratified by low versus moderate-to-high STOP-Bang-defined for obstructive sleep apnea (OSA) risk.

Variables	Moderate-to-high STOP-Bang score	Low STOP-Bang score	p
n	105	41	–
Age (years)	66.1 ± 9.5	61.2 ± 12.8	0.015
BMI (kg/m²)	30.7 ± 6.1	26.9 ± 8.0	0.002
HbA1c (%)	7.6 ± 1.6	8.0 ± 1.6	0.117
Duration of T2D (years)	15.9 ± 10.6	14.1 ± 10.4	0.409
Former smoker, n (%)	16 (15.5)	6 (15.0)	0.937
Men, n (%)	82 (78.1)	23 (21.9)	<0.001
Previous DFU, n (%)	48 (65.8)	7 (17.5)	0.010
Previous amputation, n (%)	42 (40.0)	3 (7.3)	0.021
Retinopathy, n (%)	48 (45.7)	13 (31.7)	0.123
Polyneuropathy, n (%)	68 (64.8)	19 (46.3)	0.042
Peripheral artery disease, n (%)	32 (30.5)	5 (12.2)	0.022

Values are presented as mean ± standard deviation or n (%). Information on T2D duration was available for 114 patients. BMI, body mass index; HbA1c, glycated hemoglobin; DFU, diabetic foot ulcer.

When ulcer characteristics were analyzed by STOP-Bang category, a moderate-to-high STOP-Bang-defined OSA risk was associated with older age, male predominance, and nearly a threefold higher prevalence of ulcers with moderate-to-high amputation risk according to the WIfI classification (33/61, 54.1% vs. 1/12, 8.3%; *p*=0.004) (**Table 3**). Thus, about half of the patients with a moderate-to-high risk of OSA presented with severe foot ulcer. In addition, among patients with a moderate-to-high risk of 1-year amputation, the proportion of patients with moderate-to-high STOP-Bang-defined OSA risk reaches 97.1%, significantly higher than that observed in subjects with low WIfI scores (71.8%, p=0.004) (**Figure 1**). Regarding ulcer course, in an exploratory comparison of median time to healing, healing time was markedly longer in patients with moderate-to-high STOP-Bang-defined OSA risk compared with those at low risk [120.0 (45.0-406.0) vs. 65.0 (30.0-171.0) days, p=0.032].

**Table 3:** Clinical and ulcer characteristics in patients with diabetic foot ulcer (DFU) stratified by low versus moderate-to-high STOP-Bang-defined risk for obstructive sleep apnea (OSA).

Variables	Moderate-to-high STOP-Bang score	Low STOP-Bang score	p
n	61	12	
Age (years)	67.1 ± 8.5	59.8 ± 8.1	0.007
BMI (kg/m²)	29.6 ± 5.1	31.7 ± 13.1	0.347
HbA1c (%)	7.6 ± 1.7	8.4 ± 1.1	0.144
Duration of T2D (years)	16.4 ± 10.9	13.0 ± 11.6	0.456
Former smoker, n (%)	8 (13.6)	3 (27.3)	0.251
Men, n (%)	58 (95.1)	5 (41.7)	<0.001
Previous DFU, n (%)	41 (67.2)	7 (58.3)	0.553
Previous amputation, n (%)	31 (50.7)	3 (25.0)	0.149
Retinopathy, n (%)	33 (54.1)	7 (58.3)	0.788
Polyneuropathy, n (%)	60 (98.4)	12 (100.0)	0.655
Peripheral artery disease, n (%)	25 (41.0)	4 (33.3)	0.621
Moderate-to-high WIfI score, n (%)	33 (54.1)	1 (8.3)	0.004
Time to heal (days)	120.0 (45.0-406.0)	65.0 (30.0-171.0)	0.032

Values are presented as mean ± standard deviation, median (range) or n (%). Information on T2D duration was available for 114 patients (49 with DFU and 65 controls). BMI, body mass index; HbA1c, glycated hemoglobin; DFU, diabetic foot ulcer.

Finally, in the multivariable binary logistic regression restricted to patients with DFU, the outcome was moderate-to-high 1-year amputation risk according to the WIfI classification. The model was adjusted for age, sex, HbA1c, peripheral artery disease, previous DFU, and STOP-Bang category. Moderate-to-high STOP-Bang-defined OSA risk was associated with this outcome, although with a wide confidence interval (OR 15.19, 95%CI 1.25-184.32; p=0.033) as was poorer metabolic control (HbA1c: OR 1.61, 95%CI 1.08-2.39; p=0.020) (**Table 4**). The number of patients classified as moderate-to-high WIfI amputation risk was 33/61 in the moderate-to-high STOP-Bang group and 1/12 in the low STOP-Bang group. The logistic regression model showed acceptable fit (deviance = 76.7; Nagelkerke R² = 0.376) and excellent discrimination (AUC=0.802; 95% CI 0.701-0.902)for identifying patients classified as moderate-to-high amputation risk according to WIfI (**Supplementary Figure 1**).

**Table 4:** Multivariate binary logistic multivariate regression analysis for moderate-to-high 1 year amputation risk according to the WIfI classification in patients with diabetic foot ulcer.

Predictor	OR	95%CI	P
Constant	0.001	0.05-0.66	0.037
STOP-Bang category (moderate-high vs low)	15.191	1.252-184.318	0.033
HbA1c (%)	1.606	1.078-2.391	0.020
Age (years)	1.017	0.947-1.093	0.625
Sex (female vs. male)	0.043	0.001-1.008	0.040
Peripheral artery disease (yes vs. no)	2.812	0.881-8.975	0.081
Previous DFU (yes vs. no)	0.309	0.087-1.102	0.070

HbA1c, glycated hemoglobin; T2D, type 2 diabetes; DFU, diabetic foot ulcer.

## Discussion

Our findings reveal a remarkably high prevalence of moderate-to-high STOP-Bang-defined OSA risk among patients with T2D and DFU. This expands upon the results of Maltese et al. (21), who reported a 64% prevalence of STOP-Bang scores ≥4 in a prospective cohort with both T1D and T2D, with higher scores associated with poorer ulcer healing (21). In our cohort, the prevalence of moderate-to-high OSA risk was even higher (83.5%), likely reflecting our exclusive inclusion of T2D patients, often older, with higher rates of obesity and other risk factors associated with OSA (8, 36). However, the higher unadjusted STOP-Bang score observed in patients with DFU compared with controls should be interpreted cautiously, as this difference was attenuated in sensitivity analyses accounting for sex imbalance. This is particularly relevant because male sex is one of the STOP-Bang items and was markedly more frequent in the DFU group. In addition, consistent with these observations, we found that patients with T2D and DFU at moderate-to-high OSA risk had substantially longer ulcer healing times than those at low risk. Since confirmed OSA is a well-established risk factor for cardiovascular disease, endothelial dysfunction, and impaired tissue perfusion, these underlying mechanisms could contribute to worsening lower-limb ischemia and ulcer healing in diabetes (3, 37). Therefore, we suggest that assessing OSA risk should be considered alongside other classical pathogenic factors for DFU such as male sex, older age, poor glycemic control, diabetic polyneuropathy and PAD (33, 34).

Our analysis emphasizes that moderate-to-high STOP-Bang-defined OSA risk is associated with more severe ulcer characteristics in patients with T2D and DFU, including a greater proportion of patients (>50%) at moderate-to-high 1-year amputation risk according to the WIfI classification. To our knowledge, this is the first case–control study restricted to T2D, reducing diabetes type heterogeneity present in prior reports (21, 22). Notably, we found that higher OSA risk was associated with delayed ulcer healing, one of the most important risk factors of amputation. This finding is compatible with the hypothesis that nocturnal hypoxia and its consequences may be involved in both ulcer severity and its healing. The WIfI score integrates wound extent, ischemia, and infection severity into a robust and prognostically validated tool (31). Although the time taken to complete it is slightly longer than the others, compared to other classification tools such as the Wagner or University of Texas Wound Classification Systems, WIfI is more predictive of major amputation and shows higher inter-observer agreement, justifying its use as a preferred clinical endpoint (38, 39). Nevertheless, due to the low number of amputations of our cohort we were unable to detect significant differences between those patients with moderate-to-high versus low STOP-Bang score.

Observations by Vas et al., despite involving only three patients with severe obesity, T2D, and severe untreated OSA, support the contribution of recurrent episodes of partial or complete upper-airway obstruction to impair DFU (20). Notably, CPAP initiation improved healing of DFU in two subjects, thus leading these authors to recommend actively assessing OSA in non-healing or slowly progressive cases once other contributory factors have been optimized. Our results, linking higher STOP-Bang score to elevated WIfI amputation risk, suggest that a systematic STOP-Bang-defined OSA risk assessment in DFU is useful in the management of DFU.

A plausible pathophysiological framework for the observed association between STOP-Bang-defined OSA risk, WIfI severity, and delayed healing includes recurrent intermittent hypoxia, which promotes oxidative stress, systemic inflammation, sympathetic activation, and endothelial dysfunction, leading to impaired vasodilation and reduced microvascular perfusion (11, 12, 36). Although causality cannot be established from the present study, these mechanisms may be particularly relevant in patients with DFU, in whom tissue oxygen delivery is already compromised by neuropathy, peripheral artery disease, infection, and metabolic dysregulation, and may further impair wound oxygenation and delay tissue repair (14, 31, 32). Intermittent hypoxia may also interfere with fibroblast activity, collagen deposition, angiogenesis, and immune-cell function, thereby contributing to slower healing and greater ulcer severity (1, 12, 20). More recently, Chen et al. (22) evaluated 167 Chinese DFU patients with full polysomnography, which represents a methodological strength over questionnaire-based approaches (40). Although there was no significant association between apnea-hypopnea index (AHI) and wound healing, total sleep time, sleep efficiency, and wakefulness after sleep onset were associated with wound healing. These findings suggest that sleep disturbances may also impact DFU prognosis through neurohormonal, inflammatory, neuropathic, and vascular pathways (41–45). Therefore, ulcer prognosis in patients with T2D may extend beyond hypoxemia alone, highlighting the need for future studies combining standardized ulcer risk scores (e.g., WIfI) with detailed sleep architecture analysis to unravel these complex inter-relationships.

Our results, showing an association between STOP-Bang–defined OSA risk and WIfI-based amputation risk, indicate that even simple screening tools can help flag DFU patients at higher risk for poor outcomes (22, 24, 46). However, the large odds ratio observed for STOP-Bang category should be interpreted cautiously because of the small number of patients in the low-risk STOP-Bang group and the resulting wide confidence interval, which may indicate model instability. Although several screening tools are available for OSA, STOP-Bang is widely used as a brief and practical instrument for risk stratification, particularly in clinical settings where universal polysomnography is unfeasible (30). In patients with T2D, available validation studies suggest that STOP-Bang has high sensitivity but modest specificity. For example, in a Chinese T2D population, a STOP-Bang cut-off ≥3 showed sensitivities of 85.6%, 88.6%, and 90.5% for detecting OSA defined by AHI ≥5, >15, and >30 events/hour, respectively, whereas specificity was 60.0%, 38.4%, and 27.0% across these thresholds (27). A recent meta-analysis have shown that the STOP-Bang is the most accurate brief instrument for OSA risk stratification, outperforming the Berlin questionnaire, STOP questionnaire, and Epworth Sleepiness Scale, particularly in clinical interviews and in settings with limited access to sleep clinics (29, 30, 47). Clinically, our findings underscore the potential value of incorporating OSA risk assessment by using STOP-Bang score into the routine evaluation of patients with DFUs. Given its speed, affordability, and ease of administration, the questionnaire may help prioritize patients for confirmatory sleep testing or early interventions targeting sleep quality, particularly in resource-constrained environments where universal polysomnography is unfeasible. Future prospective studies should evaluate whether confirmed OSA and targeted sleep interventions, including CPAP when clinically indicated, improve ulcer healing, disability and mortality outcomes (20).

This study has several limitations. First, STOP-Bang is useful for identifying individuals who may require confirmatory sleep assessment, but it should not be interpreted as diagnostic of OSA. In our study, no participant underwent polysomnography or sleep study confirmation as part of the protocol; consequently, some degree of misclassification is possible, particularly overestimation of true OSA prevalence due to false-positive screening results (48). In addition, because STOP-Bang includes cardiometabolic and vascular risk factors, some associations may partly reflect these overlapping risk factors rather than sleep-disordered breathing itself. Moreover, excluding patients with known OSA or CPAP treatment may also limit cohort representativeness and underestimate the overall burden of sleep-disordered breathing. Second, because STOP-Bang was administered at the first Diabetic Foot Clinic visit, after diagnosis of the active ulcer, the analyses relating OSA risk to DFU presence and WIfI severity are cross-sectional. Thus, we cannot determine whether increased OSA risk preceded DFU development or contributed causally to ulcer severity. Although time to healing was recorded during follow-up, this analysis was exploratory and should not be interpreted as evidence of causality. Third, our cohort consisted entirely of Caucasian individuals recruited from a single center, potentially limiting the generalizability of the findings to other ethnic groups or healthcare settings (7, 49). Fourth, the relatively modest sample size of DFU cohort, particularly in the subgroup of patients with low STOP-Bang-defined OSA risk, may have reduced the statistical power to detect certain associations. Accordingly, the large odds ratio observed for STOP-Bang category should be interpreted cautiously, especially given the wide confidence interval. The multivariable model should also be interpreted as exploratory, as it included 34 patients classified as moderate-to-high 1-year amputation risk according to WIfI and six candidate predictors, resulting in a modest events-per-variable ratio and potential model instability. In addition, analyses of time to ulcer healing according to STOP-Bang categories were exploratory and based on comparisons of median healing time rather than formal time-to-event methods such as Kaplan-Meier curves or Cox regression. Therefore, these analyses did not account for censoring or the full time-dependent structure of the data and should be interpreted with caution and confirmed in larger prospective cohorts. Finally, residual confounding cannot be excluded, as not all potential confounders could be included in the multivariable model because of sample-size constraints and risk of overfitting. In addition, sex imbalance between DFU cases and controls may have influenced the case-control comparison, as male sex is included in the STOP-Bang score. Despite these limitations, the study possesses notable strengths. Restricting the population to individuals with T2D minimized clinical heterogeneity and enhanced the internal validity of the findings. In addition, the use of the WIfI classification offered a standardized, prognostically relevant, and validated method for assessing limb threat and amputation risk, thereby providing a robust clinical endpoint for evaluating associations with OSA risk.

In conclusion, among patients with T2D and established DFU, higher STOP-Bang-defined OSA risk was associated with greater ulcer severity and longer healing time, although causal inference cannot be established. The initially higher STOP-Bang score observed in DFU cases compared with controls was attenuated after accounting for sex imbalance and should therefore be interpreted cautiously. These findings highlight the under-recognized role of sleep-disorders breathing risk in diabetic foot prognosis. Given its simplicity and low cost, systematic STOP-Bang screening in diabetic foot care protocols may help identify candidates for confirmatory sleep assessment and timely intervention, potentially improving limb salvage rates. Further prospective research including objective sleep assessment should evaluate whether confirmed OSA and corrective sleep interventions improve DFU outcomes and reduce amputation rates in this high-risk population.

## Data Availability

The raw data supporting the conclusions of this article will be made available by the authors, without undue reservation.
