# Supplementary material for: Obstructive sleep apnea risk and its association with diabetic foot ulcer in patients with type 2 diabetes
Source: Front Endocrinol (Lausanne). 2026 May 29;17:1813327. doi: 10.3389/fendo.2026.1813327 (PMC13259888; doi:10.3389/fendo.2026.1813327)
Supplement: Supplementary file 1 [file DataSheet1.pdf]

Supplementary Figure 1. Receiver operating characteristic (ROC) curve assessing the discriminative ability of the multivariable logistic regression model in patients with diabetic foot ulcer.

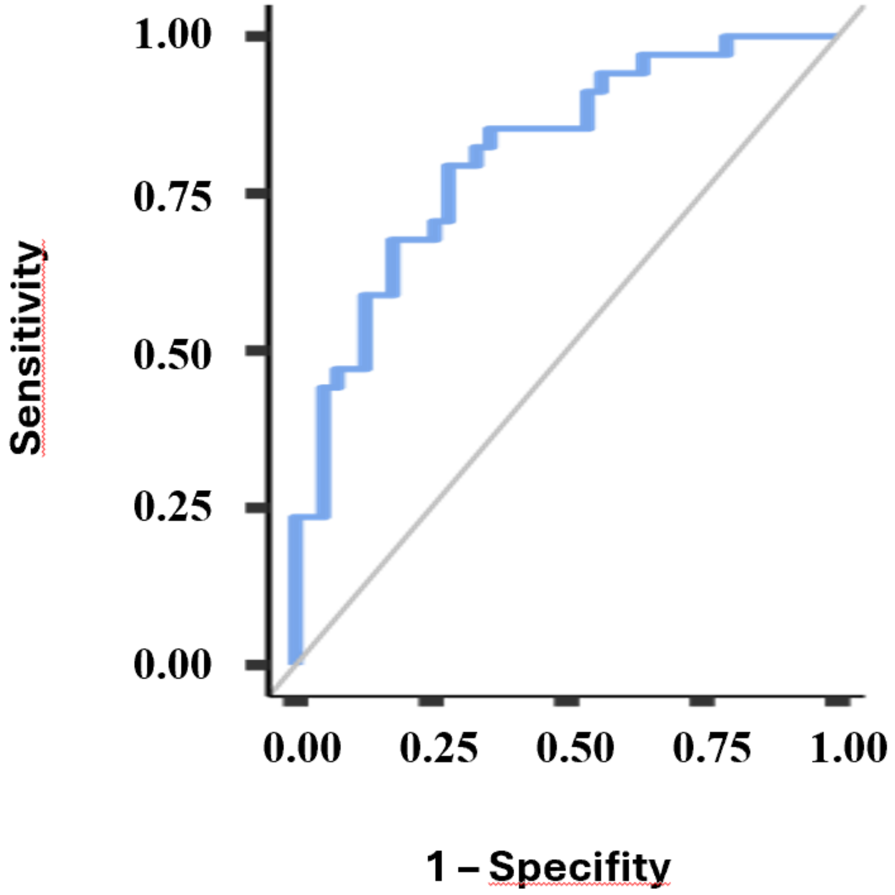

The model showed good discrimination, with an AUC of 0.802 (95% CI: 0.701–0.902), indicating robust separation between patients at low versus moderate-to-high amputation risk (WIfI classification).
