# Supplementary material for: Obstructive sleep apnea risk and its association with diabetic foot ulcer in patients with type 2 diabetes
Source: Front Endocrinol (Lausanne). 2026 May 29;17:1813327. doi: 10.3389/fendo.2026.1813327 (PMC13259888; doi:10.3389/fendo.2026.1813327)
Supplement: Supplementary file 2 [file DataSheet2.pdf]

STROBE No.	Item (short description)	Location in the manuscript
1	Title/abstract indicates study design	Abstract – Methods (“We conducted a case–control study…”)
2	Background and scientific rationale	INTRODUCTION, first paragraphs
3	Specific objectives	INTRODUCTION, last paragraph (“Therefore, the aims of the present study were…”)
4	Study design	METHODOLOGY – Design of the study and description of the study population
5	Setting, locations, and dates	Same section (Design…) and Description of patients evaluated for risk of having OSA (hospital, DF clinic, period September 2023–September 2024)
6 (a–c)	Participants: eligibility criteria, case/control selection, matching	Description of patients evaluated for risk of having OSA (cases and controls, matching)
7	Clear definition of variables (exposures, outcomes, confounders)	Main association, Specific questionnaire to assess risk of OSA, Diabetic Foot Ulcer Assessment
8	Data sources and methods of measurement	Specific questionnaire to assess risk of OSA (STOP-Bang) and Diabetic Foot Ulcer Assessment (WIfI, ischemia, infection)
9	Efforts to address potential sources of bias	Implicit in Description of patients… (criteria, matching) and explicitly in DISCUSSION – Limitations; no dedicated “Bias” subsection in Methods
10	Study size and sample size calculation	Sample size calculation
11	Handling of quantitative variables	Statistical Analysis (STOP-Bang as continuous/categorical, WIfI simplification)
12 (a–e)	Statistical methods, control for confounding, handling of matching and missing data	Statistical Analysis and paragraph on AUC-ROC; handling of matching and missing data not explicitly detailed
13 (a–c)	Participants’ flow, numbers at each stage	Described in Description of patients… (Methods) and RESULTS, first paragraph; no flowchart included

**STROBE No.**	**Item (short description)**	**Location in the manuscript**
14 (a–c)	Descriptive data (baseline characteristics, potential confounders)	RESULTS + Table 1, Table 2 (clinical characteristics, foot-related variables, complications)
15	Outcome data	RESULTS (WIfI severity, amputation risk, healing time, STOP-Bang risk)
16 (a–c)	Main results, estimates and precision, adjusted analyses	RESULTS (DFU vs controls comparisons) and Table 4 (logistic regression within DFU group)
17	Other analyses (subgroups, sensitivity analyses)	RESULTS (multivariable model, AUC-ROC); no sensitivity analyses reported
18	Key results summary	DISCUSSION, first paragraph
19	Limitations	DISCUSSION, paragraph starting with "This study has several limitations."
20	Overall interpretation in light of evidence	Rest of DISCUSSION and concluding paragraph
21	Generalisability (external validity)	DISCUSSION – Limitations (single-centre, mainly Caucasian population) and tone of the conclusion
22	Funding and role of funder	FUNDING section
